# Synthesis, Crystal Structure, and DNA-Binding Studies of a Nickel(II) Complex with the Bis(2-benzimidazolymethyl)amine Ligand

**DOI:** 10.1155/2012/609796

**Published:** 2011-06-30

**Authors:** Huilu Wu, Tao Sun, Ke Li, Bin Liu, Fan Kou, Fei Jia, Jingkun Yuan, Ying Bai

**Affiliations:** School of Chemical and Biological Engineering, Lanzhou Jiaotong University, Lanzhou 730070, China

## Abstract

A V-shaped ligand Bis(2-benzimidazolymethyl)amine (bba) and its nickel(II) picrate (pic) complex, with composition [Ni(bba)_2_](pic)_2_·3MeOH, have been synthesized and characterized on the basis of elemental analyses, molar conductivities, IR spectra, and UV/vis measurements. In the complex, the Ni(II) ion is six-coordinated with a N_2_O_4_ ligand set, resulting in a distorted octahedron coordination geometry. In addition, the DNA-binding properties of the Ni(II) complex have been investigated by electronic absorption, fluorescence, and viscosity measurements. The experimental results suggest that the nickel(II) complex binds to DNA by partial intercalation binding mode.

## 1. Introduction

Binding studies of small molecules to DNA are very important in the development of DNA molecular probes and new therapeutic reagents [1]. Transition metal complexes have attracted considerable attention as catalytic systems for use in the oxidation of organic compounds [2], probes in electron-transfer reactions involving metalloproteins [3], and intercalators with DNA [4]. Numerous biological experiments have demonstrated that DNA is the primary intracellular target of anticancer drugs; interaction between small molecules and DNA can cause damage in cancer cells, blocking the division and resulting in cell death [5–7].

 Since the benzimidazole unit is the key-building block for a variety of compounds which have crucial roles in the functions of biologically important molecules, there is a constant and growing interest over the past few years for the synthesis and biological studies of benzimidazole derivatives [8–10]. Since the characterization of urease as a nickel enzyme in 1975, the knowledge of the role of nickel in bioinorganic chemistry has been rapidly expanding [11]. The interaction of Ni(II) complexes with DNA appears to be mainly dependent on the structure of the ligand exhibiting intercalative behavior [12–14].

In this context, we synthesized and characterized a novel Ni(II) complex. Moreover, we describe the interaction of the novel Ni(II) complex with DNA using electronic absorption and fluorescence spectroscopy and viscosity measurements. 

## 2. Experimental

### 2.1. Materials and Methods

Calf thymus DNA (CT-DNA) and Ethidium bromide (EB) were purchased from Sigma Chemicals Co. (USA). All chemicals used were of analytical grade. All the experiments involving interaction of the ligand and the complexes with CT-DNA were carried out in doubly distilled water buffer containing 5 mM Tris and 50 mM NaCl and adjusted to pH 7.2 with hydrochloric acid. A solution of CT-DNA gave a ratio of UV absorbance at 260 and 280 nm of about 1.8–1.9, indicating that the CT-DNA was sufficiently free of protein [15]. The CT-DNA concentration per nucleotide was determined spectrophotometrically by employing an extinction coefficient of 6600 M^−1^ cm^−1^ at 260 nm [16]. 

Elemental analyses were performed on Carlo Erba 1106 elemental analyzer. The IR spectra were recorded in the 4000–400 cm^−1^ region with a Nicolet FT-VERTEX 70 spectrometer using KBr pellets. Electronic spectra were taken on a Lab-Tech UV Bluestar spectrophotometer. The fluorescence spectra were recorded on a 970-CRT spectrofluorophotometer. ^1^H*­*NMR spectra were obtained with a Mercury plus 400 MHz NMR spectrometer with TMS as internal standard and DMSO-*d_6_* as solvent. Electrolytic conductance measurements were made with a DDS-11A type conductivity bridge using a 10^−3^ mol·L^−1^ solution in DMF at room temperature.

### 2.2. Electronic Absorption Spectra

Absorption titration experiment was performed with fixed concentrations of the complexes while gradually increasing concentration of CT-DNA. While measuring the absorption spectra, a proper amount of CT-DNA was added to both compound solution and the reference solution to eliminate the absorbance of CT-DNA itself. From the absorption titration data, the binding constant (*K*_*b*_) was determined using [17]

(1) $$\frac{\left[\text{DNA}\right]}{\varepsilon_{a}-\varepsilon_{f}}=\frac{\left[\text{DNA}\right]}{\varepsilon_{b}-\varepsilon_{f}}+\frac{1}{K_{b}\left(\varepsilon_{b}-\varepsilon_{f}\right)},$$

where [DNA] is the concentration of DNA in base pairs, the apparent absorption coefficient, *ε*_*a*_, *ε*_*f*_, and *ε*_*b*_ correspond to *A*_obsd_/[*M*], the extinction coefficient of the free compounds and the extinction coefficient of the compound when fully bound to DNA, respectively. In plots of [DNA]/(*ε*_*a*_ − *ε*_*f*_) versus [DNA], *K*_*b*_ is given by the ratio of slope to the intercept.

### 2.3. Fluorescence Spectra

EB emits intense fluoresence in the presence of CT-DNA due to its strong intercalation between the adjacent CT-DNA base pairs. It was previously reported that the enhanced fluorescence can be quenched by the addition of a second molecule [18]. The extent of fluorescence quenching of EB bound to CT-DNA can be used to determine the extent of binding between the second molecule and CT-DNA. The competitive binding experiments were carried out in the buffer by keeping [DNA]/[EB] = 1 and varying the concentrations of the compounds. The fluorescence spectra of EB were measured using an excitation wavelength of 520 nm and the emission range was set between 550 and 750 nm. The spectra were analyzed according to the classical Stern-Volmer equation [19],

(2) $$\frac{I_{0}}{I}=1+K_{\text{sv}}\left[Q\right],$$

where *I*_0_ and *I* are the fluorescence intensities at 599 nm in the absence and presence of the quencher, respectively, *K*_sv_ is the linear Stern-Volmer quenching constant, [*Q*] is the concentration of the quencher.

### 2.4. Viscosity Measurements

Viscosity experiments were conducted on an Ubbelohde viscometer, immersed in a thermostated water-bath maintained at 25.0 ± 0.1°C. DNA samples approximately 200 bp in average length were prepared by sonicating in order to minimize complexities arising from DNA flexibility [20]. Titrations were performed for the compounds (3 mM), and each compound was introduced into the CT-DNA solution (50 *μ*M) present in the viscometer. Data were presented as (*η*−*η*_0_)^1/3^ versus the ratio of the concentration of the compound to CT-DNA, where **η** is the viscosity of CT-DNA in the presence of the complex, and *η*_0_ is the viscosity of CT-DNA alone. Viscosity values were calculated from the observed flow time of CT-DNA containing solutions corrected from the flow time of buffer alone (*t*_0_), *η* = (*t* − *t*_0_)/*t*_0_.

### 2.5. Synthesis

**Scheme 1:**
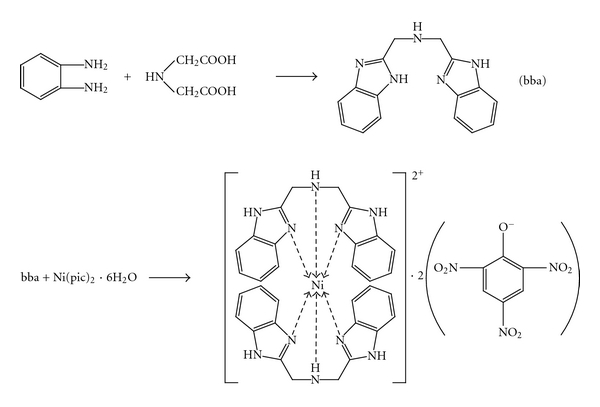
The synthesis of ligand bba and its Ni(II) complex.

 The synthetic route for the ligand bba and its Ni(II) complex are shown in Scheme 1.

#### 2.5.1. Bis(2-benzimidazolymethyl)amine (bba)

The ligand bba was synthesized according to the procedure reported by Berends and Stephan [21]. The infrared spectra and UV spectra of the bba were almost consistent with the literature. Elemental analysis: C_16_H_15_N_5_ (Mr = 277.33 g·mol^−1^) calcd: C 69.30; H 5.45; N 25.26%; found: C 69.35; H 5.47; N 25.16%. IR (KBr, pellet, cm^−1^): 1270s (*ν*_C–N_), 1620s (*ν*_C=N_), UV-vis (*λ*, nm): 277, 283, *ε*_277_ = 5.99 × 10^2^ L·mol^−1^·cm^−1^, *ε*_283_ = 5.73 × 10^2^ L·mol^−1^·cm^−1^. ^1^HNMR (DMSO-*d*_6_, 300 MHz) *δ*: 12.3 (1H, N-H); 7.144 (m, 4H); 7.5 (d, 4H); 4.0 (s, 4H). Λ_M_ (DMF, 297 K): 1.29 S·cm^2^·mol^−1^.

#### 2.5.2. [Ni(bba)_2_](pic)_2_·3MeOH

The ligand bba (0.4 mmol) and Ni(II) picrate (0.2 mmol) were dissolved in methanol (15 mL). A blue-green crystalline product which formed rapidly was filtered off, washed with methanol and absolute Et_2_O, and dried in vacuo. The dried precipitate was dissolved in DMF resulting in a blue-green solution that was allowed to evaporate at room temperature. Blue-green crystals suitable for X-ray diffraction studies were obtained after one week. C_47_H_36_N_16_Ni O_17_ (Mr = 1155.63 g·mol^−1^) calcd: C 48.85; H 3.14; N 19.39%; found: C 48.79; H 3.16; N 19.53%. IR (KBr, pellet, cm^−1^): 1272s (*ν*_C–N_), 1434 (*ν*_C=N–C=C_), 1487s (*ν*_C=N_), UV-vis (*λ*, nm): 275, 280, 407, *ε*_275_ = 6.55 × 10^2^ L·mol^−1^·cm^−1^, *ε*_280_ = 6.50 × 10^2^ L·mol^−1^·cm^−1^, *ε*_407_ = 7.99 × 10^2^ L·mol^−1^·cm^−1^. Λ_M_ (DMF, 297 K): 128.5 S·cm^2^·mol^−1^.

### 2.6. Crystal Structure Determination

A suitable single crystal was mounted on a glass fiber and the intensity data were collected on a Bruker Smart CCD diffractometer with graphite-monochromated Mo K*α* radiation (*λ* = 0.71073 Å) at 296 K. Data reduction and cell refinement were performed using the SMART and SAINT programs [22]. The structure was solved by direct methods and refined by full-matrix least squares against *F* ^2^ of data using SHELXTL software [23]. All H atoms were found in different electron maps and were subsequently refined in a riding-model approximation with C–H distances ranging from 0.95 to 0.99 Å. Basic crystal data, description of the diffraction experiment, and details of the structure refinement are given in Table 1.

## 3. Results and Discussion

The ligand bba and its Ni(II) complex are very stable in the air. They are remarkably soluble in polar solvents such as DMF, DMSO, and MeCN; slightly soluble in ethanol, methanol, ethyl acetate, and chloroform. The molar conductivities in DMF solution indicate that bba (1.29 S·cm^2^·mol^−1^) is nonelectrolyte compound and its Ni(II) complex is 1 : 2 electrolyte compound [24].

### 3.1. Spectral Characterization

In the bba ligand, a strong band is found at ca. 1270 cm^−1^ together along with a broad band at 1436 cm^−1^. By analogy with the assigned bands of imidazole, the former can be attributed to **ν**(C=N–C=C), while the latter can be attributed to **ν**(C=N) [25–27]. One of them shift to the higher frequency by around 41 cm^−1^ in the complex, which implies direct coordination of all three imine nitrogen atoms to metal ions. This is the preferred nitrogen atom for coordination as found for other metal complexes with benzimidazoles [28]. Information regarding the possible bonding modes of the picrate and benzimidazole rings may also be obtained from the IR spectra, such as 709, 744, 1272, 1363, 1434, 1487, and 1633 cm^−1^ [29]. This fact agrees with the result determined by X-ray diffraction.

DMF solutions of ligand bba and its complexes show, as expected, almost identical UV spectra. The UV bands of bba (275, 280 nm) are only marginally blue shifted (1-2 nm) in the complexes, which is clear evidence of C=N coordination to the metal ions center. The absorption bands are assigned to **π*→* π* (imidazole) transitions. The bands of picrate (407 nm) are assigned to**π**→π* transitions.

### 3.2. Crystal Structure of [Ni(bba)_2_](pic)_2_·3MeOH

**Figure 1:**
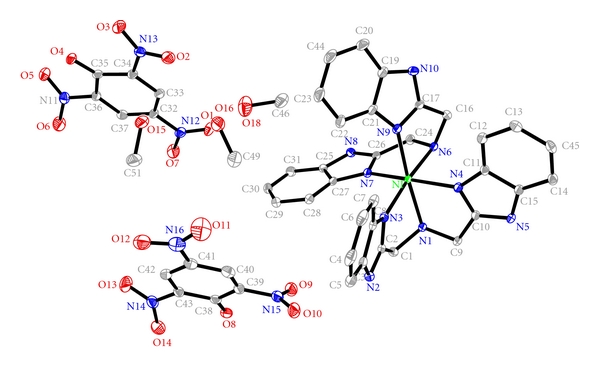
The molecular structure of the Ni(II) complex showing displacement ellipsoids at the 30% probability level. Hydrogen atoms have been omitted for clarity.

The molecular structure of the Ni(II) complex is shown in Figure 1, selected bond lengths and angles are summarized in Table 2. The Ni(II) atom is six-coordinate with a NiN_4_O_2_ environment. The bba ligand acts as a tridentate N-donor and O-donor. The coordination geometry of the Ni(II) may be best described as distorted octahedral with four coordination nitrogen atoms from an ideal equatorial plane. The maximum deviation (N9) from the plane containing these four N atoms is 0.764 Å. The bond average length between the Ni ion and the apical N atom (N1, N6) is 2.171 Å, which is about 0.097 Å longer than the bond average length between the Ni ion and four coordination N atoms from an equatorial plane. This geometry is assumed by the Ni(II) to relieve the steric crowding. Therefore, compared with a regular octahedron, it reflects a relatively distorted coordination octahedron around Ni(II).

### 3.3. Spectral Studies of the Interactions with DNA

#### 3.3.1. Electronic Absorption Titration

**Figure 2:**
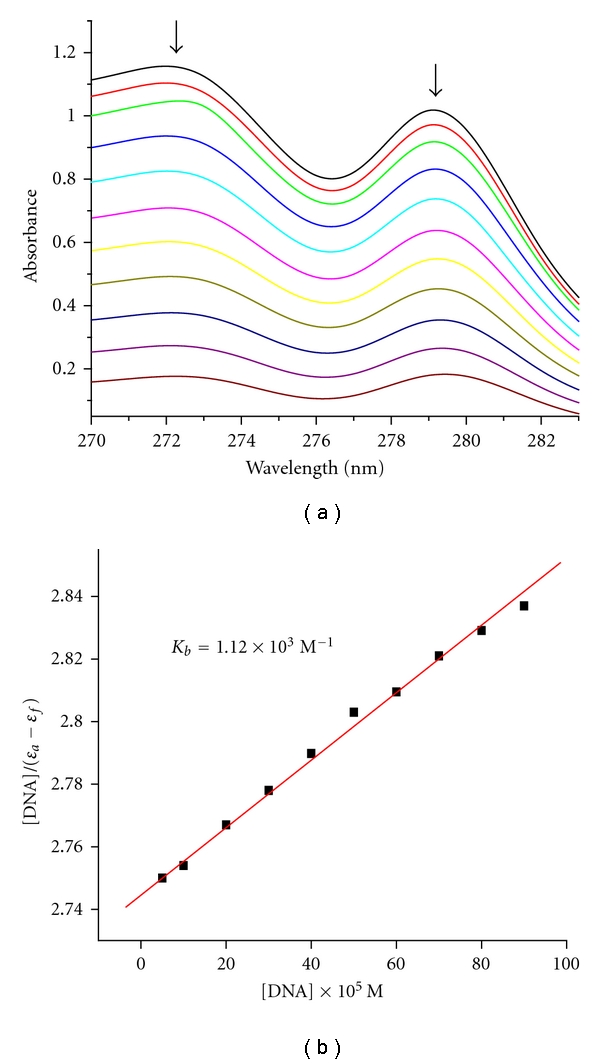
Electronic spectra of the Ni(II) complex (30 *μ*M) in the presence of 0, 5, 10, 20, 30, 40, 50, 60, 70, 80, and 90 *μ*L CT-DNA. [DNA] = 2.5 × 10^−5^ M. Arrow shows the absorbance changes upon increasing CT-DNA concentration. Plots of [DNA]/(*ε*_*a*_ − *ε*_*f*_) versus [DNA] for the titration of the Ni(II) complex with CT-DNA.

Electronic absorption spectroscopy is universally employed to determine the binding characteristics of metal complexes with DNA [30–32]. The absorption spectra of the Ni(II) complex in the absence and presence of CT-DNA are given in Figure 2. There are two well-resolved bands at about 272, 278 nm for the complex. The **λ** for the ligand increases only from 272 to 273, and for the complex from 278 to 279 nm, a slight red shift about 1 nm under identical experimental conditions. The slight red shift suggests that the Ni(II) complex interacts with DNA [33]. 

The binding constant *K*_*b*_ for the complex has been determined from the plot of [DNA]/(*ε*_*a*_ − *ε*_*f*_) versus [DNA] and was found to be 1.12 × 10^3^ M ^−1^. Compared with those of the so-called DNA-intercalative ruthenium complexes (1.1 × 10^4^–4.8 × 10^4^ M^−1^) [34], the binding constants (*K*_*b*_) of the Ni(II) complex suggest that the complex with DNA with an affinity is less than the classical intercalators.

#### 3.3.2. Fluorescence Spectroscopic Studies

**Figure 3:**
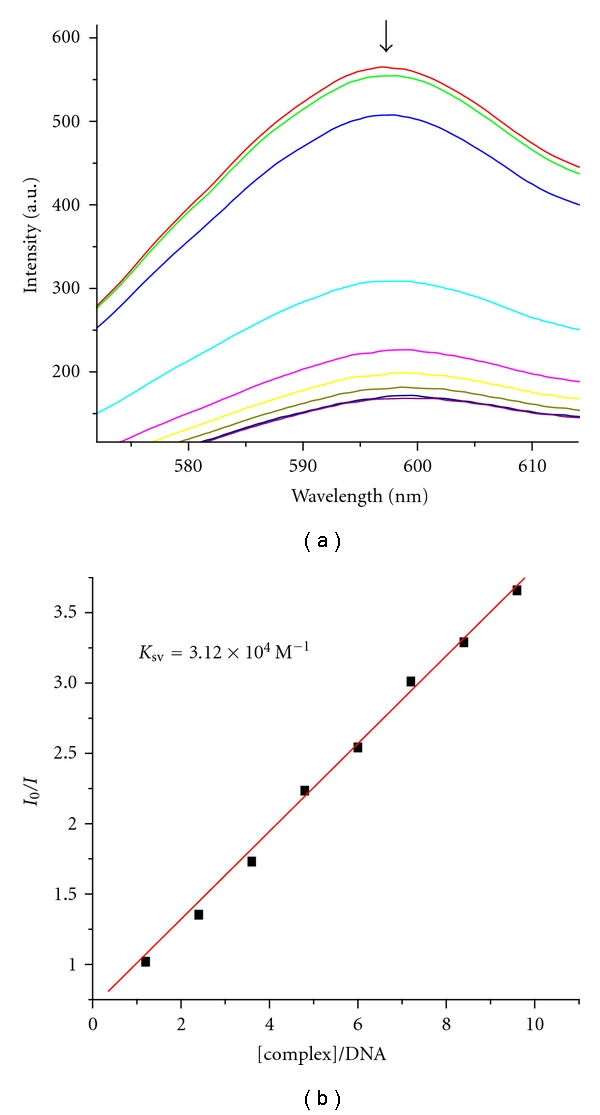
Emission spectra of EB bound to DNA in the presence of the complex. [Complex] = 3 × 10^−3^ M; *λ*_ex_ = 520 nm. The arrow shows the intensity changes upon increasing concentrations of the complex. Fluorescence quenching curves of EB bound to CT-DNA by the Ni(II) complex. (Plots of *I*_0_/*I* versus [Complex]/DNA.).

In general, measurement of the ability of a complex to affect the EB fluorescence intensity in the EB-DNA adduct allows determination of the affinity of the complex for DNA, whatever the binding mode may be. If a complex can replace EB from DNA-bound EB, the fluorescence of the solution will be quenched due to the fact that free EB molecules are readily quenched by the surrounding water molecules [35]. For all the compounds, no emission was observed either alone or in the presence of CT-DNA in the buffer. The fluorescence quenching of EB bound to CT-DNA by the Ni(II) complex is shown in Figure 3. The quenching of EB bound to CT-DNA by the Ni(II) complex is in good agreement with the linear Stern-Volmer equation, which provides further evidence that the Ni(II) complex bind to DNA. The quenching plots illustrate that the quenching of EB bound to DNA by the complex is in good agreement with the linear Stern-Volmer equation, which also proves that the complex binds to DNA. The *K*_sv_ value for the Ni(II) complex is 3.12 × 10^4^ M^−1^. The data suggest that the Ni(II) complex interacts with DNA.

#### 3.3.3. Viscosity Studies

**Figure 4:**
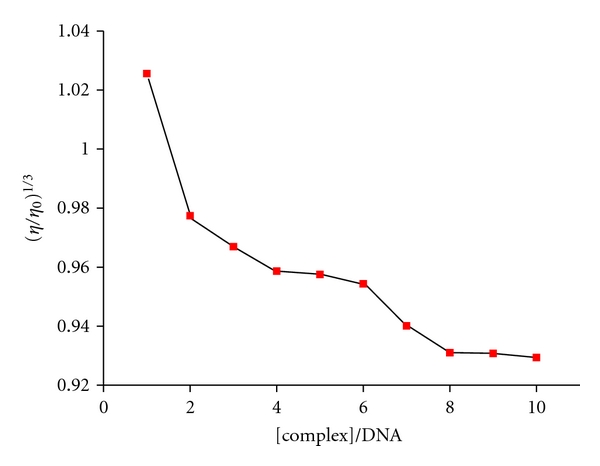
Effect of increasing amounts of the Ni(II) complex on the relative viscosity of CT-DNA at 25.0 ± 0.1°C.

Optical photophysical techniques are widely used to study the binding model of the ligand, metal complexes, and DNA but not to give sufficient clues to support a binding model. Therefore, viscosity measurements were carried out to further clarify the interaction of metal complexes and DNA. Hydrodynamic measurements that are sensitive to the length change (i.e., viscosity and sedimentation) are regarded as the least ambiguous and the most critical tests of a binding model in solution in the absence of crystallographic structural data [15, 20]. A classical intercalative mode causes a significant increase in viscosity of DNA solution due to increase in separation of base pairs at intercalation sites and hence an increase in overall DNA length. By contrast, complexes that bind exclusively in the DNA grooves by partial and/or nonclassical intercalation, under the same conditions, typically cause less pronounced (positive or negative) or no change in DNA solution viscosity [20]. The values of (*η* − *η*_0_)^1/3^ were plotted against [compound]/[DNA] (Figure 4). For the Ni(II) complex, as increasing the amounts of compound, the viscosity of DNA decreases steadily. The decreased relative viscosity of DNA may be explained by a binding mode which produced bends or kinks in the DNA and thus reduced its effective length and concomitantly its viscosity. The results suggest that the Ni(II) complex may bind to DNA by partial intercalation. 

## 4. Conclusions

In this paper, a new Ni(II) complex has been synthesized and characterized. Moreover, the DNA-binding properties of the Ni(II) complex were investigated by electronic absorption, fluorescence, and viscosity measurements. The experimental results indicate that the Ni(II) complex can bind to CT-DNA by partial intercalation mode. Information obtained from our study will be helpful to understand the mechanism of interactions of benzimidazoles and their complexes with nucleic acids and should be useful in the development of potential probes of DNA structure and conformation.

## Figures and Tables

**Table 1:** Crystallographic data and data collection parameters for the Ni(II) complex.

Complex	[Ni(bba)_2_](pic)_2_·3MeOH
Molecular formula	C_47_H_36_N_16_NiO_17_
Molecular weight	1155.63
Crystal system	Triclinic
Space group	P-1
a (Å)	10.4758 (9)
b (Å)	16.1097 (13)
c (Å)	17.2302 (14)
*α* (°)	107.5590 (10)
*β* (°)	107.5880 (10)
*γ* (°)	96.9150 (10)
*V *(Å^3^)	2570.1 (4)
*Z*	2
*ρ*_cald_ (mg m^−3^)	1.493
Absorption coefficient (mm^−1^)	0.467
*F* (000)	1188
Crystal size (mm)	0.41 × 0.38 × 0.31
**θ** range for data collection (°)	2.04–25.00
*h/k/l* (max, min)	−12, 12/−16, 19/−20, 20
Reflections collected	18579
Independent reflections	8974 [*R*(int) = 0.0203]
Data/restraints/parameters	8974/6/746
Goodness-of-fit on *F* ^2^	1.097
Final *R*_1_, *wR*_2_ indices [*I* > 2*σ*(*I*)]	0.0383, 0.1135
*R*_1_, *wR*_2_ indices (all data)	0.0466, 0.1194
Largest differences peak and hole (eÅ^−3^)	0.734 and −0.384

**Table 2:** Selected bond lengths (Å) and angles (deg) of the Ni(II) complex.

Bond lengths					
Ni–N(1)	2.1647 (19)	Ni–N(4)	2.0793(18)	Ni–N(6)	2.1788(19)
Ni–N(3)	2.0899 (19)	Ni–N(7)	2.0667 (18)	Ni–N(9)	2.0628 (19)
Bond angles					
N(1)–Ni–N(6)	94.12 (7)	N(9)–Ni–N(7)	173.40 (7)	N(9)–Ni–N(4)	98.11 (7)
N(3)–Ni–N(7)	173.40 (7)	N(3)–Ni–N(1)	79.29 (7)	N(7)–Ni–N(4)	166.55 (7)
N(9)–Ni–N(3)	107.52 (7)	N(7)–Ni–N(3)	98.95 (7)	N(3)–Ni–N(4)	89.98 (7)
N(1)–Ni–N(9)	173.19 (7)	N(7)–Ni–N(1)	90.23 (7)	N(1)–Ni–N(4)	81.52 (7)
N(9)–Ni–N(6)	79.07 (8)	N(7)–Ni–N(6)	81.11 (7)	N(4)–Ni–N(6)	88.87 (7)

## Appendix

### Additional Data

CCDC 825141 contains the additional crystallographic data for this paper. These data can be obtained free of charge from The Cambridge Crystallographic Data Centre via http://www.ccdc.cam.ac.uk/data_request/cif.

## References

[1] Mrksich M, Dervan PB (1993). Antiparallel side-by-side heterodimer for sequence-specific recognition in the minor groove of DNA by a distamycin/1-methylimidazole-2-carboxamide-netropsin pair. *Journal of the American Chemical Society*.

[2] Kokubo C, Katsuki T (1996). Highly enantioselective catalytic oxidation of alkyl aryl sulfides using Mn-salen catalyst. *Tetrahedron*.

[3] Schoumacker S, Hamelin O, Pécaut J, Fontecave M (2003). Catalytic asymmetric sulfoxidation by chiral manganese complexes: acetylacetonate anions as chirality switches. *Inorganic Chemistry*.

[4] Dupureur CM, Barton JK (1997). Structural Studies of Λ- and Δ-[Ru(phen)_2_dppz]^2+^ Bound to d(GTCGAC)_2_: characterization of Enantioselective Intercalation. *Inorganic Chemistry*.

[5] Hemmert C, Pitié M, Renz M, Gornitzka H, Soulet S, Meunier B (2001). Preparation, characterization and crystal structures of manganese(II), iron(III) and copper(II) complexes of the bis[di-1,1-(2-pyridyl)ethyl] amine (BDPEA) ligand; evaluation of their DNA cleavage activities. *Journal of Biological Inorganic Chemistry*.

[6] Li VS, Choi D, Wang Z, Jimenez LS, Tang M, Kohn H (1996). Role of the C-10 substituent in mitomycin C-1-DNA bonding. *Journal of the American Chemical Society*.

[7] Zuber G, Quada JC, Hecht SM (1998). Sequence selective cleavage of a DNA octanucleotide by chlorinated bithiazoles and bleomycins. *Journal of the American Chemical Society*.

[8] Gellis A, Kovacic H, Boufatah N, Vanelle P (2008). Synthesis and cytotoxicity evaluation of some benzimidazole-4,7-diones as bioreductive anticancer agents. *European Journal of Medicinal Chemistry*.

[9] Güven ÖÖ, Erdoğan T, Göker H, Yildiz S (2007). Synthesis and antimicrobial activity of some novel phenyl and benzimidazole substituted benzyl ethers. *Bioorganic and Medicinal Chemistry Letters*.

[10] Kopańska K, Najda A, Zebrowska J (2004). Synthesis and activity of 1*H*-benzimidazole and 1*H*-benzotriazole derivatives as inhibitors of Acanthamoeba castellanii. *Bioorganic and Medicinal Chemistry*.

[11] Skyrianou KC, Perdih F, Turel I, Kessissoglou DP, Psomas G (2010). Nickel-quinolones interaction—part 2: interaction of nickel(II) with the antibacterial drug oxolinic acid. *Journal of Inorganic Biochemistry*.

[12] Skyrianou KC, Raptopoulou CP, Psycharis V, Kessissoglou DP, Psomas G (2009). Structure, cyclic voltammetry and DNA-binding properties of the bis(pyridine)bis(sparfloxacinato)nickel(II) complex. *Polyhedron*.

[13] Jin Y, Lewis MA, Gokhale NH, Long EC, Cowan JA (2007). Influence of stereochemistry and redox potentials on the single- and double-strand DNA cleavage efficiency of Cu(II)-and Ni(II)*·*Lys-Gly-his-derived ATCUN metallopeptides. *Journal of the American Chemical Society*.

[14] Bisceglie F, Baldini M, Belicchi-Ferrari M (2007). Metal complexes of retinoid derivatives with antiproliferative activity: synthesis, characterization and DNA interaction studies. *European Journal of Medicinal Chemistry*.

[15] Chaires JB (1993). Tris(phenanthroline)ruthenium(II) enantiomer interactions with DNA: mode and specificity of binding. *Biochemistry*.

[16] Marmur J (1963). A procedure for the isolation of deoxyribonucleic acid from microorganisms. *Methods in Enzymology*.

[17] Wolfe A, Shimer GH, Meehan T (1987). Polycyclic aromatic hydrocarbons physically intercalate into duplex regions of denatured DNA. *Biochemistry*.

[18] Chauhan M, Banerjee K, Arjmand F (2007). DNA binding studies of novel copper(II) complexes containing L-tryptophan as chiral auxiliary: in vitro antitumor activity of Cu-Sn_2_ complex in human neuroblastoma cells. *Inorganic Chemistry*.

[19] Lakowicz JR, Weber G (1973). Quenching of fluorescence by oxygen. A probe for structural fluctuations in macromolecules. *Biochemistry*.

[20] Satyanarayana S, Dabrowiak JC, Chaires JB (1992). Neither Δ- nor Λ-tris(phenanthroline)ruthenium(II) binds to DNA by classical intercalation. *Biochemistry*.

[21] Berends HP, Stephan DW (1984). Copper(I) and copper(II) complexes of biologically relevant tridentate ligands. *Inorganica Chimica Acta*.

[22] Bruker (2000). *Smart Saint and Sadabs*.

[23] Sheldrick GM (1996). *SHELXTL*.

[24] Geary WJ (1971). The use of conductivity measurements in organic solvents for the characterisation of coordination compounds. *Coordination Chemistry Reviews*.

[25] Su CY, Kang BS, Du CX, Yang QC, Mak TCW (2000). Formation of mono-, bi-, tri-, and tetranuclear Ag(I) complexes of *C*
_3_-symmetric tripodal benzimidaxole ligands. *Inorganic Chemistry*.

[26] Sundberg RJ, Martin RB (1974). Interactions of histidine and other imidazole derivatives with transition metal ions in chemical and biological systems. *Chemical Reviews*.

[27] McKee V, Zvagulis M, Reed CA (1985). Further insight into magnetostructural correlations in binuclear copper(II) species related to methemocyanin: X-ray crystal structure of a 1,2-*μ*-nitrito complex. *Inorganic Chemistry*.

[28] Lane TJ, Nakagawa I, Walter JL, Kandathil AJ (1962). Infrared investigation of certain imidazole derivatives and their metal chelates. *Inorganic Chemistry*.

[29] Wu H, Yun R, Li K, Wang K, Huang X, Sun T (2009). Synthesis, crystal structure and spectra properties of the nickel (II) complex with 1,3-bis(1-benzylbenzimidazol2-yl)-2-oxopropane. *Synthesis and Reactivity in Inorganic, Metal-Organic and Nano-Metal Chemistry*.

[30] Li H, Le XY, Pang DW, Deng H, Xu ZH, Lin ZH (2005). DNA-binding and cleavage studies of novel copper(II) complex with L-phenylalaninate and 1,4,8,9-tetra-aza-triphenylene ligands. *Journal of Inorganic Biochemistry*.

[31] Vaidyanathan VG, Nair BU (2003). Synthesis, characterization, and DNA binding studies of a chromium(III) complex containing a tridentate ligand. *European Journal of Inorganic Chemistry*.

[32] Vaidyanathan VG, Nair BU (2004). Nucleobase oxidation of DNA by (terpyridyl)chromium(III) derivatives. *European Journal of Inorganic Chemistry*.

[33] Liu J, Zhang T, Lu T (2002). DNA-binding and cleavage studies of macrocyclic copper(II) complexes. *Journal of Inorganic Biochemistry*.

[34] Pyle AM, Rehmann JP, Meshoyrer R, Kumar CV, Turro NJ, Barton JK (1989). Mixed-ligand complexes of ruthenium(II): factors governing binding to DNA. *Journal of the American Chemical Society*.

[35] Baguley BC, Le Bret M (1984). Quenching of DNA-ethidium fluorescence by amsacrine and other antitumor agents: a possible electron-transfer effect. *Biochemistry*.

